# Blockchain and cloud computing-based secure electronic healthcare records storage and sharing

**DOI:** 10.3389/fpubh.2022.938707

**Published:** 2022-07-19

**Authors:** Amna Amanat, Muhammad Rizwan, Carsten Maple, Yousaf Bin Zikria, Ahmad S. Almadhor, Sung Won Kim

**Affiliations:** ^1^Department of Computer Science, Kinnaird College for Women, Lahore, Pakistan; ^2^Secure Cyber Systems Research Group, WMG, University of Warwick, Coventry, United Kingdom; ^3^Department of Information and Communication Engineering, Yeungnam University, Gyeongsan, South Korea; ^4^College of Computer and Information Sciences, Jouf University, Sakakah, Saudi Arabia

**Keywords:** blockchain, cloud computing, electronic healthcare records, decentralized, Internet of Things

## Abstract

Healthcare information is essential for both service providers and patients. Further secure sharing and maintenance of Electronic Healthcare Records (EHR) are imperative. EHR systems in healthcare have traditionally relied on a centralized system (e.g., cloud) to exchange health data across healthcare stakeholders, which may expose private and sensitive patient information. EHR has struggled to meet the demands of several stakeholders and systems in terms of safety, isolation, and other regulatory constraints. Blockchain is a distributed, decentralized ledger technology that can provide secured, validated, and immutable data sharing facilities. Blockchain creates a distributed ledger system using techniques of cryptography (hashes) that are consistent and permit actions to be carried out in a distributed manner without needing a centralized authority. Data exploitation is difficult and evident in a blockchain network due to its immutability. We propose an architecture based on blockchain technology that authenticates the user identity using a Proof of Stake (POS) cryptography consensus mechanism and Secure Hash Algorithm (SHA256) to secure EHR sharing among different electronic healthcare systems. An Elliptic Curve Digital Signature Algorithm (ECDSA) is used to verify EHR sensors to assemble and transmit data to cloud infrastructure. Results indicate that the proposed solution performs exceptionally well when compared with existing solutions, which include Proof-Of-Work (POW), Secure Hash Algorithm (SHA-1), and Message Digest (MD5) in terms of power consumption, authenticity, and security of healthcare records.

## 1. Introduction

**Figure 1:**
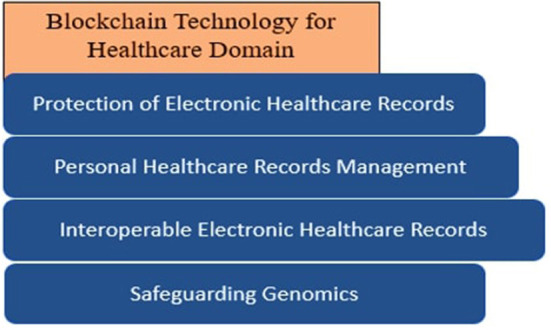
Healthcare features needs blockchain security.

Healthcare records require security and privacy protection during storage and sharing (1–7). Traditionally data sharing systems use centralized systems, which can present a major point of failure (8). Several blockchain variations have been presented since the launch of bitcoin about a decade ago (9). One of the sectors that are gaining traction with blockchain is the health industry (2). Figure 1 illustrates various applications of blockchain in the healthcare domain.

Technological advancements such as the Internet of Things (IoT), Industrial IoT, and big data have aided the expansion and innovation of healthcare worldwide and the development of smart healthcare systems (10, 11).

Smart healthcare often incorporates EHR and cloud data that combine these EHR with mobile IoT by using IoT and communication devices for the development of medical and health services and the improvement of administrative services (12–14). Although the smart healthcare business has made rapid progress, there are still security issues (15, 16). Blockchain technology provides features such as decentralization, Peer-to-Peer (P2P) networking architecture, secrecy, tamper-evidence, and auditability, which can be effective for data sharing, transactions, and supply-chain management.

**Figure 2:**
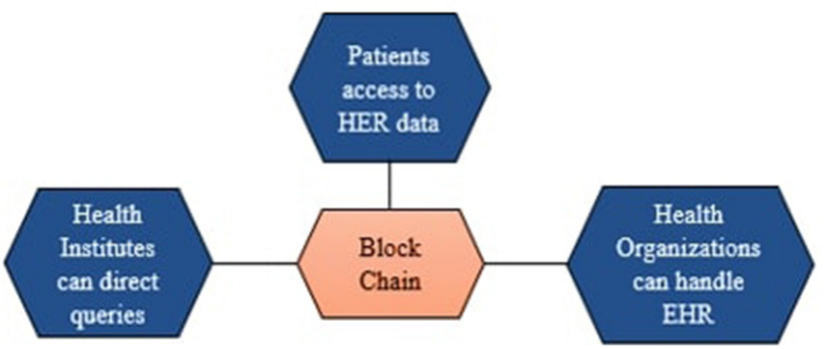
Blockchain uses for EHR.

The blend of blockchain and E-health can solve several challenges traditional healthcare solutions face like information sharing, data security, and privacy protection and improve user-centered smart healthcare solutions, as shown in Figure 2. Blockchain has gained the interest of entire business corporations because data saved on the blockchain is highly trustworthy and easily accessible through duplication. However, the research on the use of blockchain in the healthcare sector is insufficient (17).

Most of the existing studies on the blockchain are related to the preservation of information system security, sustainable supply chain platforms in smart healthcare, blockchain-based healthcare monitoring systems, and tracking of operational environment on medical products *via* blockchain (18). The implications of blockchain in digital healthcare are difficult to explain as it encompasses all aspects of the sector. Blockchain is being promoted as a valuable tool for maintaining critical confidential data, especially in the healthcare, clinical science, health sciences, and insurance industries. Secrecy and security breaches in the healthcare profession are rising every year. When the data is available on distributed systems, lack of privacy and data hacking are the main threats to the security of healthcare records. Blockchain technology provides several measures to secure healthcare system records using cryptography tools and techniques. Blockchain helps secure sensitive medical information like drug prescriptions, pregnancy reports, and supply-chain data from cyber-attacks (19).

Based on previous research, security is the major problem to be solved. The development of advanced hardware and the rise of new technologies such as cloud computing, 5G, blockchain, and Artificial Intelligence (AI) create new values and improve the quality of life based on the existing services. Despite that, when technology advanced, then the threats and limitations also took place (20). AI technologies help to centralize power inside the hands of a small number of companies that can acquire and process enormous volumes of data, while blockchain can be used for secure transfer of EHR of each individual and encourage interoperability of data (21). As most of the data is handled by giant corporations, a private blockchain is not transparent enough, and its use is restricted. This study governs the conformation of healthcare records using blockchain technology. The framework used in this work is a blockchain-based smart healthcare system that uses the alliance chain method for transaction-level tasks to deliver data records to users or stakeholders for lowering communication cost, lowering computation cost, lowering risks, and increasing trust in blockchain technology (22–24).

We propose a framework that provides the benefits of blockchain for secure and efficient sharing and management of electronic health records using smart contracts. The intelligent contract, which is dependent on medical record management and is limited by the system, is a specific blockchain application in smart healthcare. We implement the framework using the SHA256 algorithm. We choose blockchain for interoperable EHR sharing, and storage (25).

Healthcare systems tend to be plagued by problems that frequently result in increased costs or declining health results (disease and death). Based on previous studies, the critical challenge to be solved is security. Blockchain technology has been promoted as a beneficial tool for managing data confidentiality, secrecy, and security, particularly in healthcare. Blockchain provides security to healthcare records using cryptography techniques and protects them from data breaching. Therefore, this research is based on the secure, reliable, and authenticated sharing of EHR among different sources.

### 1.1. Contributions of this paper

This paper makes the following contributions:

- We propose a blockchain-based framework to secure and authenticate the health sector records sharing system.
- The main objective of our work is to protect the EHR and secure the sharing and storage of EHR on the cloud using blockchain.
- We use the POS consensus technique with the SHA256 algorithm to generate hash values to authenticate data integrity and the ECDSA technique for records verification. We use the sensor to collect the data and then store the sensor data on cloud computing-based storage to make data modifications impenetrable.
- We present a better healthcare security model for the user and the administration with its practical implementation using JavaScript class by enabling “nmp module,” which provides secure and authenticated records sharing capabilities.
- The evaluation results show that the proposed framework provides high security while sharing and storing the EHR.

### 1.2. Organization of the paper

The rest of the part is formed in an accompanying way. We discuss the literature review in Section 2. Then, we discuss the proposed methodology in Section 3. The practical implementation of proposed approaches, the detailed results, and the discussion are presented in Section 4. Finally, the paper is concluded in Section 5 with the future work.

## 2. Literature review

**Figure 3:**
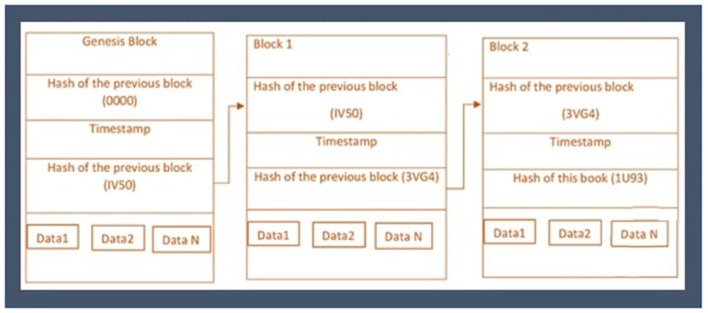
Healthcare blockchain ecosystem.

The use of the blockchain for secure sharing of EHR and its other applications in healthcare systems has been illustrated in many previous studies. Several hospitals are involved in various proposed blockchain-based works (26). Secinaro et al. (27) described the usage and benefits of blockchain in the fields of auditing, accounting, and business management. Siyal et al. (28) introduced a Healthcare Data Gateway (HDG) framework, which is one of the famous examples of personal clinical data monitoring using a private blockchain. Li et al. (29) proposed cross enterprises and distributed ecosystem using edge computing and blockchain for secure sharing of knowledge and services for manufacturers. Pandey and Litoriya (25) presented the blockchain-based DASH application developed using the Decentralized applications of blockchain for secure and safe healthcare records sharing. Griggs et al. (30) developed a blockchain application based on Ethereum for safe monitoring of patients living far away using sensors. Siyal et al. (28) proposed the solution, Personal health record (PHR) for patients based on a public blockchain to easily access and share their health records as represented in Figure 3.

Similarly, Chen et al. (31) proposed a blockchain-based cloud application to store and share the healthcare data of patients. In this framework, patients have access to share and manage their health records without the interference of any intermediary. Wang et al. (32) proposed a blockchain-based artificial intelligence system that helps the patients decide on treatment and doctors to examine the overall process of treatment. Shen et al. (33) proposed a framework of MedChain for secure sharing of healthcare data in sessions based on the techniques of digest blockchain and cryptography.

Jiang et al. (34) proposed BloCHIE based on an off-chain mechanism to maintain validity and privacy during sharing of electronic medical records and personal healthcare data. Pandey and Litoriya (25) proposed a framework based on attribute-based signature and MA-ABS to maintain privacy and security during the Electronic healthcare information exchange of patients. Shubbar et al. (35) proposed the DermoNet framework to assist and check the patients of dermatology. Abid et al. (36) proposed a blockchain-based distributed framework to provide secure patient examining facilities by storing patients' histories and prescriptions on the cloud. Zhang et al. (37) proposed a secure and efficient data storage and sharing scheme for blockchain-based mobile-edge computing using signature private key sharing technique to realize data and backups for cloud data storage.

Authors in (38) proposed an efficient, large-scale batch verification method using ECDSA. Authors in (39) proposed an authentication protocol for Wireless Sensor Medical Networks (WMSN) using blockchain and physically unclonable functions to protect the network from security and vulnerability attacks. Authors in (39) proposed a novel pairing-free certificateless scheme based on blockchain technology for the maintenance of data privacy and security of IIOT devices. Zhang et al. (40) provided a detailed survey on blockchain applications in various fields used for the security and protection of data.

Research on blockchain for healthcare management is increasing and still needs more attention and research. Blockchain applications have also been implemented in oncology, biomedical research to store DNA, insurance fraud detection of healthcare departments, anti-counterfeiting drug details to store medicines records, and pharmaceutical supply-chain management of healthcare medicines. From the related work, it is identified that blockchain has exceptional capabilities in the medical field. However, the difficulties must be applied to healthcare administration that can be beneficial to link different systems and improve the accuracy and safety of EHR (25). We analyze from the literature review that most of the research papers presented theoretical frameworks or models regarding EHR management using blockchain. However, technical details are missing, and existing proposed frameworks have limitations against data security, bandwidth, immaturity, and privacy of blockchain technology in healthcare because the algorithms used previously, such as POW, MD5, and SHA-1 is less efficient, costly, and consumes more computation power and communication cost.

## 3. Proposed framework

**Figure 4:**
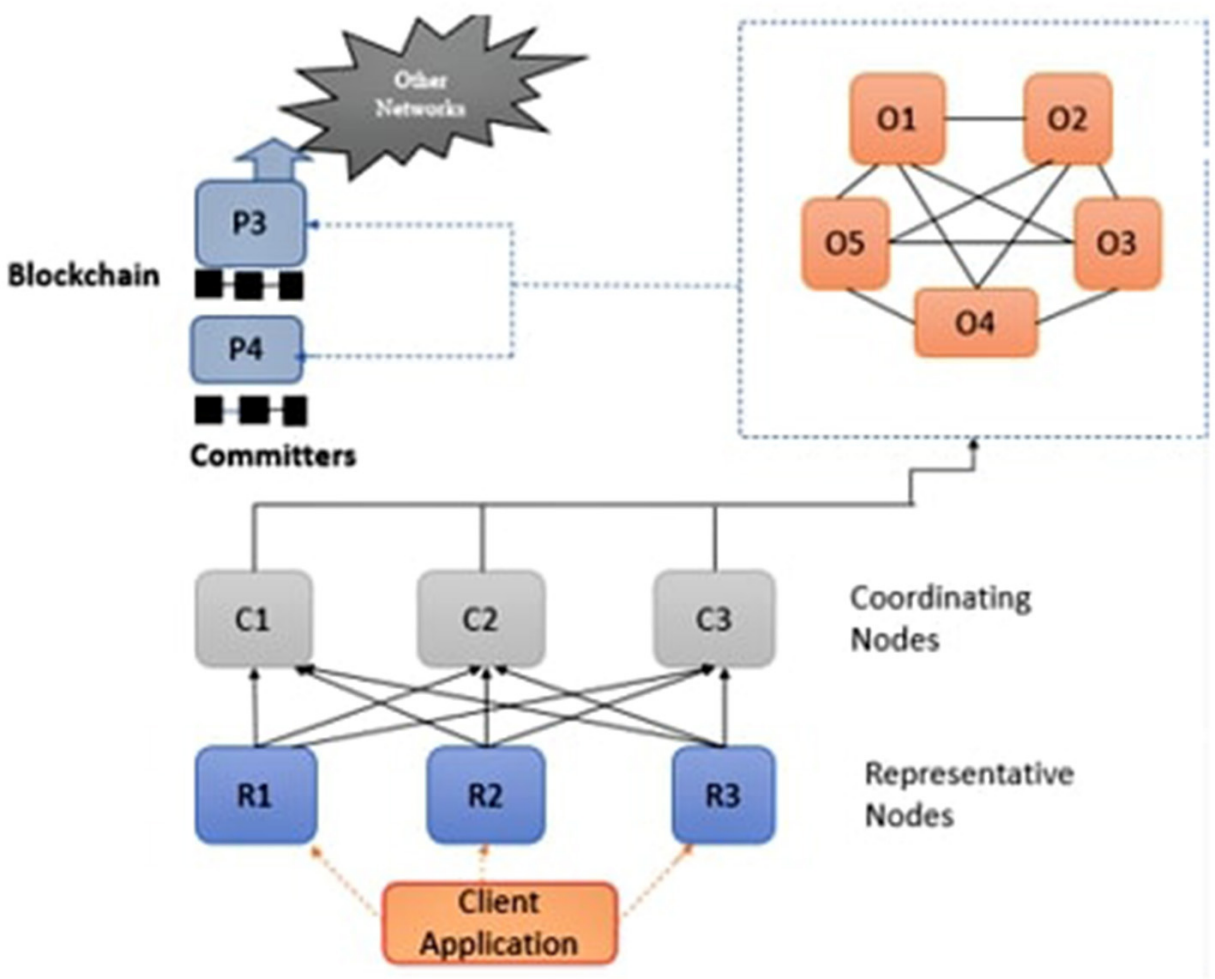
Blockchain architecture in healthcare domain.

We propose a secure peer-to-peer and decentralized framework for all healthcare providers and patients to maintain and manage demanding healthcare data. The proposed framework is based on a cryptographic POS consensus mechanism. The main reason to use blockchain is because of its property of immutability. Hyperledger builds confidential smart contracts among two parties using a POS consensus algorithm to exchange healthcare data, which improves data privacy by isolating transactions and consumes less energy and computation power. The design of the proposed framework is represented in Figure 4. The framework provides secured sharing and storage of EHR among individuals and stakeholders.

The framework is decentralized, consisting of multiple nodes that can participate in the network to perform secure healthcare data sharing. The proposed framework is essentially safe from failure and data loss. The nodes in the proposed framework establish a ledger of the previous transaction using the blockchain technique of hashing, SHA256 cryptographic hash function, compute hash, and nodes need enough disk space to store hashes of all previous and next transactions. The secure hashing algorithm is implemented for decentralized blockchain (41).

**Figure 5:**
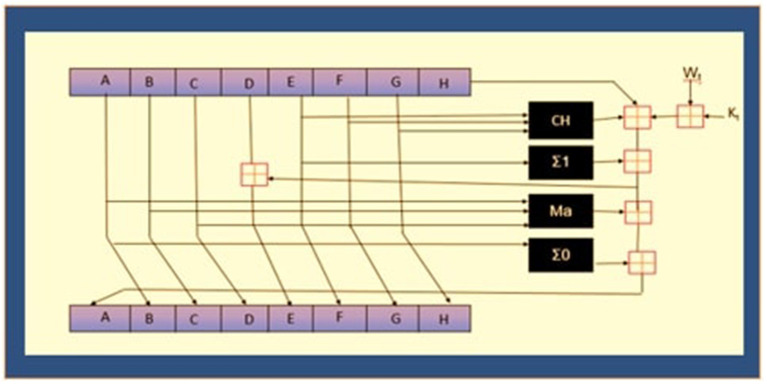
Hash function computation process.

For the computation of hash values as represented in Figure 5, modular exponential technique were used (41, 42). This approach calculates the exponent (e) of power nth, which is divided by the modulus of a non-negative integer (n), and the remainder of an integer base value (b).

The hash value is also generated by using the concept of Trapdoor One-way functions as described in Equation (1), which are defined between two sets like X and Y (43).


(1) $$\begin{array}{r} \begin{array}{c} f:a\Rightarrow b,with\;f\left(a\right)=b \end{array} \end{array}$$

Where *f* is a trapdoor function hiding some secret information inside it, and the function of hashing is described in Equation (2).


(2) $$\begin{array}{r} \begin{array}{c} h:M\Rightarrow {0,1}^{n},with\;h\left(m\right)=m \end{array} \end{array}$$

The Algorithm 1 converts the message into binary codes by using American Standard Code for Information Interchange (ASCII). Padding of zeros in the code gives the correct size of the block (512 bits), then the block is divided into 16 groups starting from *M*_0_–*M*_15_, *h* shows hash values, and *w* represents the words in each group of the block.

**Algorithm 1:** SHA256 Crypto Computation

1: Increase the number of zero 0 bits in the data input until it reaches 448 bits, then add a 64-bit length to the supplied data until it reaches 512 bits
2: Divide the 512-bit data into 16 groups after it has been merged :*M*_0_–*M*_15_
3: Set up the vectors *K*_0_–*K*_63_ and *h*_0_–*h*_7_
4: Let A,B,C,D,E,F,G, and H have the following initial values: *h*_0_–*h*_7_ **Computation**
5: Set t to loop from 0 to 63, then update as follows: *B*_*t*+1_=*A*_*t*_,
6: *C*_*t*+1_ = *B*_*t*_, *D*_*t*+1_ = *C*_*t*_, *F*_*t*+1_ = *E*_*t*_, *G*_*t*+1_ = *F*_*t*_, *H*_*t*+1_ = *G*_*t*_, *A*_*t*+1_ = *H*_*t*_+Σ_1_(*E*_1_)+*Ch*(*E*_*t*_, *F*_*t*_, *G*_*t*_)+*K*_*t*_+*W*_*t*_+Σ_0_(*A*_*t*_)+*Maj*(*A*_*t*_, *B*_*t*_, *C*_*t*_), *E*_*t*+1_+*H*_*t*_+Σ_1_(*E*_*t*_)+*Ch*(*E*_*t*_, *F*_*t*_, *G*_*t*_)+*K*_*t*_+*W*_*t*_+*D*_*t*_,
7: **Let** *h*_0_ = *h*_0_+*A*_63_, *h*_1_ = *h*_1_+*B*_63_, *h*_2_+*C*_63_, *h*_3_ = *h*_3_+*D*_63_, *h*_4_ = *h*_4_+*E*_63_, *h*_5_ = *h*_5_+*F*_6_3, *h*_6_ = *h*_6_+*G*_63_, *h*_7_ = *h*_7_+*H*_63_ ***h*_0_−*h*_7_**.
8: Σ_1_(*E*_*t*_), Σ_0_(*A*_*t*_), *Maj*(*A*_*t*_, *B*_*t*_, *C*_*t*_) and *Ch*(*E*_*t*_, *F*_*t*_, *G*_*t*_) are logical functions, and *W*_*t*_ is updated according to:
$W_{t}=\{\begin{array}{ll} M_{0}, & \$\text{0}\leq \text{t}\leq \text{15}\$,\\ \sigma_{1}(W_{t-2})+W_{t-7}+\sigma_{0}(W_{t-15}) & \\ +W_{t-16}, & \$\text{16}\leq \text{t}\leq \text{63}\$ \end{array}$

In the blockchain, the mining Algorithm 2 is used to merge new transactions B with previous transaction blocks P using block hash(H). This method helps to maintain a record of previous transactions, which is helpful for the validation and computation process of the transaction. Cloud mining is used to store the EHR data in the cloud.

**Algorithm 2:** Blockchain Mining Pseudocode

1: P: The hash of the previously mined block
2: B : A block of transaction
3: H : A hash function
4: D : Difficulty level
5: 0 Retrieve P
6: Modify B
7: IF H(P, B, Some Random Number) ≥ D End
8: GOTO 1

For verification of hashes, the elliptic curve technique is used, which is based on the Weierstrass Equation (3).


(3) $$\begin{array}{r} y^{2}=x^{3}+ax+b \end{array}$$

Block-chain uses ECDSA for the verification of ledger data entered by the users or participants. ECDSA is a cryptographic algorithm that ensures that data is shared among authorized nodes only because it depends on hash value. To add the previous block's hash with the upcoming block's hash, the following Equation (4) has been used. The terms used in the equations show hash blocks containing secret information.


(4) $$\begin{array}{r} \begin{array}{c} b=\left(x2-y2\right)/\left(x1-y1\right)mod\\ Mt1=b2-y1-x1modM\\ t2=b\left(y1-t1\right)-x2modM \end{array} \end{array}$$

There are five separate nodes participating in consensus in the network, including visitors, coordinators, agents, administrators, and the contributor nodes. The visitor nodes request the required services from the network. Agent nodes manage client node requests and provide them authentication services before approval. Coordinator nodes confirm the approval and acknowledge or refuse the request for further processing. Administrator nodes obtain authenticated requests from several coordinator nodes, manage transactions into frames, and transmit the freshly produced frames to neighbor coordinators and contributors, who circulate them further throughout the network. Using this approach, the updated state is initiated among the entire network.

The block or frame comprises a block header and a transaction. The timestamp, current block hash, preceding block hash, and Merkle root are included in the block header. Merkle root calculates the current and previous blocks' hash value because it is more efficient and secure and takes less space. Hence this process form chain of blocks. Every contributing network device must give a regional, updated blockchain version according to a framework.The transactions are tamper-resistant as a result of this setup. When an attacker manipulates a transaction in his favor, the Merkle root, which represents all of the candidate transaction's hash, is altered, and the hash for that block is altered as well. As the targeted chunk is likewise connected to its next chain using a hash, there will be a point of conflict, and the system will not accept this alteration. As a result, the entire network contributes to assuring the confidentiality and reliability of the transactions. An Authentication and Authorization Agency (AAA) identifies every individual connected to the network. AAA guarantees that only certified entities carry out their responsibilities by the allowed access (25). The following section outlines a plan for the safe and secure storage of EHR.

We presume that the healthcare specifics include a variety of hospitals, surgeons, and other personnel records. Special recognition keys are used to identify healthcare specifics, including hospitals, doctors, personnel, and patients. Medications, diagnostic reports, bills, insurance policies, and birth and death records are examples of healthcare transactions made and shared regularly. A block is 30 KB in size, with a maximum of 15 transactions. There is also a 6-min time limit, and this means that even if a building block only includes one transaction, it will be completed in the blockchain after that period. Transactions might no longer wait for an unknown time to complete. Those who are permitted can initiate or investigate transactions on the network, which is closed. Patients' data can be kept private by eliminating personal details, such as names and contact information, or by employing an internal investigation procedure to ensure that the rationale for utilizing patient data is valid and authentic (44). A hash of the patient ID issued by AAA is used to incorporate privacy into the network. Only this hash is used to record and recognize every content on the network. As a result of hashing's one-way nature, the patient's details cannot be tracked by glancing at E-documents. This information must be stored in the distributed ledger once a document is constructed or transferred. A visitor notifies the agents of such operations, and each agent node validates the transaction's source and rights. Steps 1 through 4 describe the reliability, authenticity, and validation of transactions by ensuring that the message has not been changed during passing from one block to another.

**Step 1:** Assume that an ECG operator is doing an ECG of a patient having ID 0001. Before submitting this transaction to the agent nodes, the visitor node (ECG operator) would sign it with his private key. The agent nodes verify the visitor's validity and authorizations before approving or rejecting the proposal with an “A” or “R” indicator. Agent nodes submit suggestions to coordinators for approval or rejection.

**Step 2:** The coordinators make sure that the new visitor's proposal gets the necessary approvals and permissions in accordance with the acceptance or sanctioning policy. If a visitor proposal violates the policy, coordinators do not forward transactions to administrators. The coordinators also do the additional task of using smart contracts to validate the transactions. A smart contract, for instance, would examine if this transaction is valid “ECG film made for patient Id 1bcd9cefbc5ef8afn5ad4d4f f74b3ade at /hospital/report-store/ECG/1bcd9cefbc5ef8afn5ad4d4ff 74b3ade-05-04-2021-1600.ecg” is confirmed by looking it up in the ECG database: look it up (“/hospital/report-store/ECG /1bcd9cefbc5ef8afn5ad4d4ff74b3ade-05-04-2021-1600.ecg”). The action is confirmed if the search results in a declaration. Patients and healthcare providers can also sign smart contracts with jointly agreed-upon clauses and conditions.

**Step 3:** The administrator then receives the transactions from coordinators, orders them according to their logs, and uses the consensus mechanism to produce a new block of transactions. Rather than owning the distributed ledger, administrators regularly hold a backup of the last block hash.

**Step 4:** Administrators then transmit the freshly devised frame or block of the transaction to each of the coordinating and contributing nodes, which authenticate the transactions within the block once more before executing and updating their ledgers. This secondary validation ensures that a transaction has not been invalidated in the interim. The suggested framework architecture incorporates several unique characteristics that make it robust, durable, business-oriented, accessible, and private:

1. Only the contributing nodes are responsible for maintaining the public ledger in the proposed structure, while coordinators may also preserve ledger copies. Administrators only keep a copy of the most recent block hash, not the entire ledger. This allows for the use of a basic organizing service to serve many organizations or intermediary organizing services to serve numerous organizations without compromising the confidentiality and privacy of previous data.
2. The agent nodes perform the duties of a custodian by determining whether a transaction is approved and originated from a reliable source—this aids in the early application of business logic by preventing incorrect transactions from progressing through the system.
3. The use of hash values for collecting and processing health information enables privacy while also allowing the system to be transparent. For example, transactions are public to everyone who has a copy of the ledger, but nobody can determine the patient by glancing at the transaction.
4. In the proposed approach, a contributor node may be a member of another hierarchical organizational system and transmit the new blocks to other neighbors. As a result, the system is robust and fair from the start.
5. High reliability is provided through distributed consensus for administrators. For instance, a one-node reliable system can be developed using four nodes.
6. Early identification of finite-state anomalies by implementing smart contracts overall coordinating nodes prevents non-deterministic transactions from propagating farther into the organizing service.
7. Writing regulations that restrict the number of agents and coordinators who can approve and validate transactions could help to speed up the process. Concurrently, a more significant number of requests can be accepted from visitors and forwarded to the administration panel. Conventional systems store patient demographics, allergies, medications, and large documents such as MRIs, X-rays, and endoscopic results off the chain. Authorized individuals can access them *via* secure links.

## 4. Results and discussions

The proposed solution is tested on 12 Windows Ten (10) 64-bit Desktops having 4 Gb Of ram and a Hp 2.4-GHz CPU. A crypto module in node.js computes the 256-bit SHA-2 hash code. 64-bit hexadecimal digits are used to generate the 256-bits hash value. Here, the block is created with an introductory JavaScript class consisting of 12 nodes divided into two groups of three nodes identified as coordinating and authoritative nodes correspondingly. One node within the coordinating set keeps a local copy of the master ledger. The responsibility of ordering services has been given to five of the remaining nodes for managing the public ledger, and two nodes operate as contributors and one as a visitor. JavaScript has been used on some ports to test the validation and authentication, e.g., authoritative node listening to some messages on port 7777, and node.js implementation is represented as shown in Algorithm 3.

**Algorithm 3:** SHA256 Module: Crypto Node.js Source Code

1: var crypto=require('crypto');
2: exports.sha256Hash function (pubkey)
3: return crypto.createHash ('sha256'). update (pubkey).digest ('hex');

### 4.1. Performance evaluation

We use transaction throughput and computation time as evaluation metric to evaluate the performance of proposed framework.The throughput is the rate at which valid transactions are committed by the network in specified period of time.

We performed analysis by performing transactions using proposed methodology on various nodes. We analyzed the throughput time spending on communication and completing a transaction using the proposed framework.

The Algorithms 3, 4 show the source code used for the implementation process of transaction commitment and its status of approval or refusal during the information exchange between several nodes. To analyze the performance, we perform an analysis based on the throughput of various participating nodes' transactions for the proposed blockchain framework. Then we analyzed the implementation of network throughput on different configuration nodes, which results are explained in Table 1.

**Algorithm 4:** Source code of Authoritative Node.js

1: var hash=require(‘./sha256module'),
2: var sys require(“sys”),
3: myhttp require(“http”)
4: myhttp.createServer(function(request,response)
5: sys.puts(“Proposal Received”);
6: message request.body.message
7: pubkey request.body.patientId
8: signature request.body.signature
9: source request.body.source
10: if (sourcehash.sha256Hash(pubkey)&& verifySig(signature))
11: var-proposal(message:message,flag: 'A')
12: request.body.message proposal
13: request.redirect (‘…/coordinator1');
14: request.redirect (‘…/coordinator2');
15: (‘…/coordinator3')
16: else
17: var proposal (message:message,flag: 'R')
18: request.body.message proposal
19: request.redirect (‘…/coordinator1');
20: request.redirect (‘…/coordinator2');
21: request.redirect (‘…/coordinator3');
22: response.writeHeader(200, “Content-Type”: “text/plain”);
23: response.write(“Proposal Received”);
24: response.end();
25: .listen(7777);

**Table 1:** Network throughout with numerous configured nodes.

**Conf. #**	**Authoritative**	**Coordinating**	**Ordering**	**Contributors**	**Throughout**
3	2	2	1	3	3
5	3	3	5	1	6
3	3	3	4	2	6
4	3	3	3	2	7
5	3	3	2	2	9
6	3	3	1	2	10
7	3	2	5	2	6
8	3	1	5	2	7
9	2	3	5	2	5
10	1	3	5	2	5
11	2	3	4	2	6
12	2	3	3	2	8
13	2	3	2	2	9
14	2	3	1	2	12
15	1	3	3	2	9
16	1	3	3	2	9
17	2	2	3	1	9
18	1	1	1	1	16

In Table 1, the same values show the results of constant parameter for every configuration node means secure sharing without any alteration in data, and different values show the results of changed configuration parameter values which indicates that if data has been altered during the interim process, then it gives the changed output every time. The amount of transactions every 6 min is used to determine the throughput.

**Figure 6:**
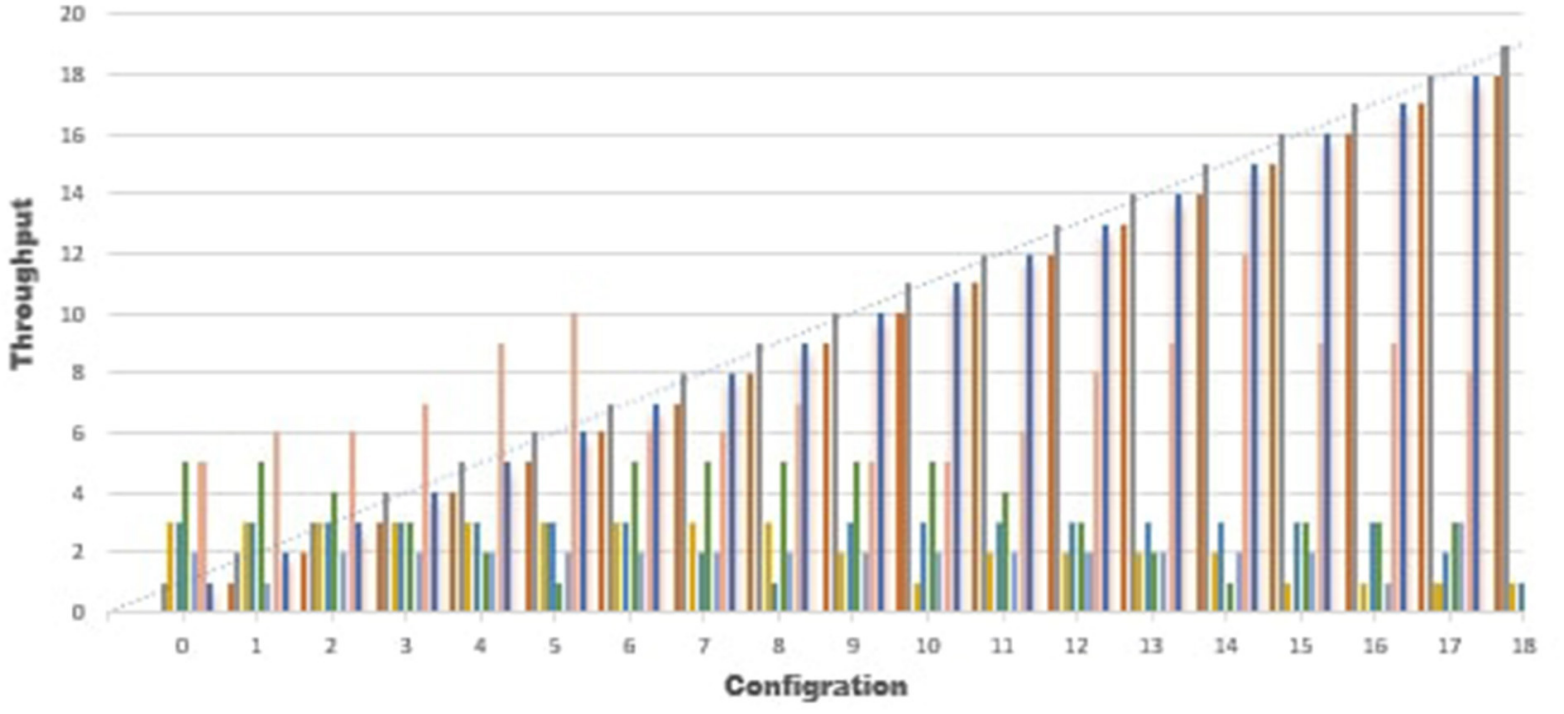
Analysis of performance.

**Figure 7:**
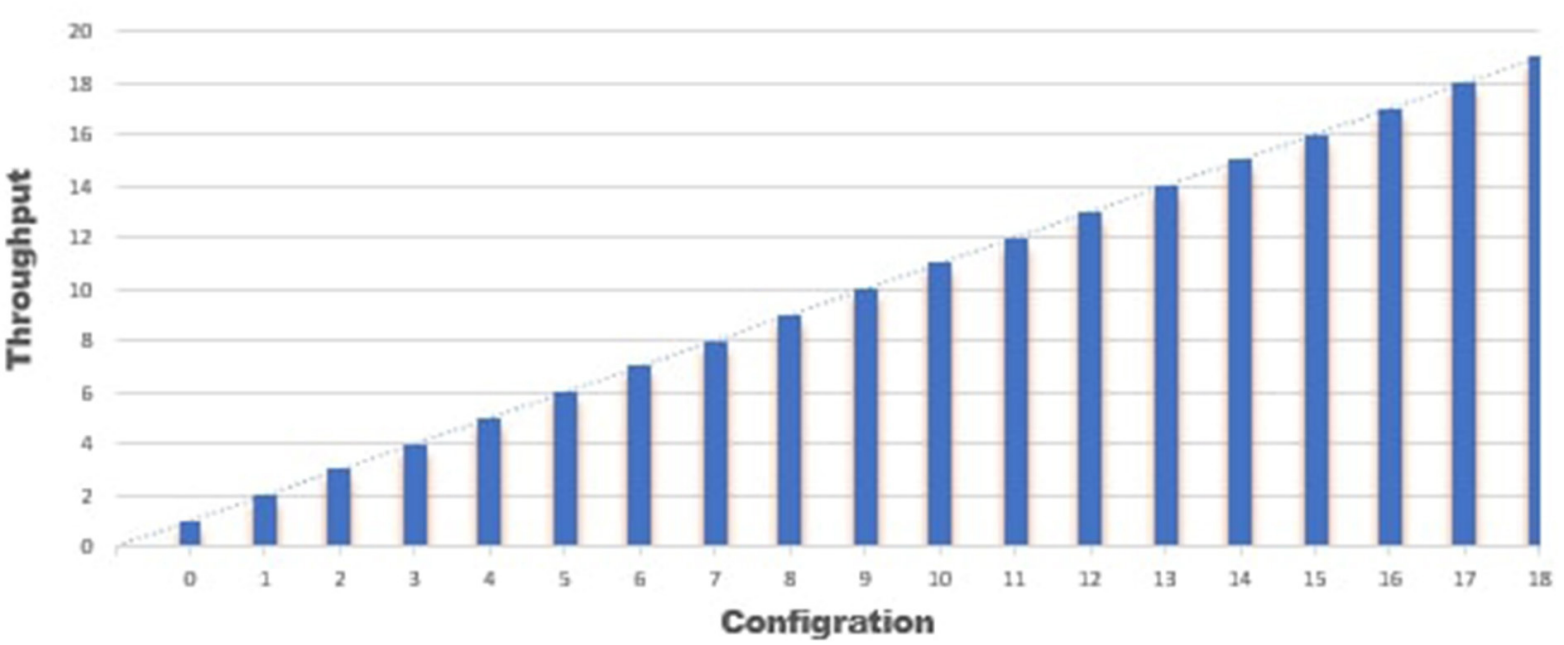
Performance analysis.

The Graphs in Figures 6, 7 show the results of configuration nodes, whose implementation details are given in Table 1. Most ordering sites significantly impact system throughput, whereas authoritative contributors and coordinators have a minor impact due to network latency. We also discovered that up to 17 operations could be completed with the lowest load. We also compare the performance of the proposed framework with existing approaches and found better performance in terms of throughput, computation cost, communication cost and secure storage and sharing.

## 5. Conclusion

We proposed a translucent, secure, and distributed framework for secure healthcare data exchange using blockchain technology. Blockchain provides robust, traceable, and unchangeable data sharing and storage facilities relative to traditional healthcare systems. We proposed a blockchain-based framework to store patients' confidential details using patients' hash IDs in distributed hyperledger using POS consensus with crypto SHA256. For the verification of records and secure transactions, ECDSA has been used. We used the nmp module of the JavaScript class to implement the proposed framework. Only recognized users can perform transactions and get stored data through this framework. Due to the storage issues on the blockchain, we stored the data on the conventional server using sensors and transmitted the sensor data into a cloud computing framework. As the transaction backups are stored on various nodes in the network, if any node is found malicious, it will not harm the network's data. We compared the results of the proposed solution with existing frameworks and found that the proposed solution performs better. We can conclude that the SHA256 and ECDSA can provide security, integrity, and authenticity in various medical data sharing and storing mechanisms. The framework has some limitations related to the storage capacity. In the future, this technique can be made more robust with the integration of federated learning to achieve extensive protection (45, 46). This work can also be adapted for a trustable courier system and monitoring corruption intolerance.

## Data availability statement

The original contributions presented in the study are included in the article/supplementary material, further inquiries can be directed to the corresponding author/s.

## Author contributions

AAm and MR: conceptualization. YZ: data curation. AAm: formal analysis, investigation, and methodology. SK: funding acquisition. SK and MR: project administration. AAm and AAl: resources. CM and MR: software, validation, and writing—review and editing. AAm and SK: supervision. YZ and AAl: visualization. All authors contributed to the article and approved the submitted version.

## Funding

This research was supported in part by Basic Science Research Program through the National Research Foundation of Korea (NRF) funded by the Ministry of Education (NRF-2021R1A6A1A03039493) and in part by the NRF grant funded by the Korea Government (MSIT) (NRF-2022R1A2C1004401).

## Conflict of interest

The authors declare that the research was conducted in the absence of any commercial or financial relationships that could be construed as a potential conflict of interest.

## Publisher's note

All claims expressed in this article are solely those of the authors and do not necessarily represent those of their affiliated organizations, or those of the publisher, the editors and the reviewers. Any product that may be evaluated in this article, or claim that may be made by its manufacturer, is not guaranteed or endorsed by the publisher.
